# The Pan American Medical Congress

**Published:** 1892-09

**Authors:** 


### The Pan-American Medical Congress.

CINCINNATI, O., SEPT. 2, 1892.

In accordance with the wishes of the Committee on Permanent Organization Drs. I. N. Love, A. B. Richardson, L. S. McMurtry, R. B. Hall, T. V. Fitzpatrick and Chas. A. L. Reed met in Cincinnati, O., and signed the legal form of application for the Articles of Incorporation of the Pan-American Medical Congress, which articles of incorporation were duly issued by the Secretary of the State of Ohio under date of March 15th, A. D. 1892.

There are twenty-one sections in the congress, each devoted to some specialty in medicine. Number 19 is that of Oral and Dental Surgery, with Drs. M. H. Fletcher, of Cincinnati, as Chairman, and John S. Marshall, of Chicago, as Secretary.

The honorary chairmen of this section are:

DR. J. TAFT, Cincinnati,
DR. H. J. MCKELLOPS, St. Louis,
DR. R. R. ANDREWS, Boston,
DR. WM. CARR, New York,
DR. E. A. BALDWIN, Chicago,
DR. B. H. CATCHING, Atlanta,
DR. FRANCIS PEABODY, Louisville,
DR. J. H. HATCH, San Francisco,

Dr. Louis Jack, Philadelphia,
Dr. A. O. Hunt, Iowa City,
Dr. S. B. Brown, Ft. Wayne,
Dr. Geo. J. Fredericks, New Orleans,
Dr. J. C. Storey, Dallas,
Dr. J. B. Willmot, Toronto, Canada,
Dr. Geo. Beers, Montreal.

"The members of the Congress shall consist of such members of the medical profession of the western hemisphere, including the West Indies and Hawaii, as shall comply with the special regulations regarding registration, or who shall render service to the Congress in the capacity of foreign officers."

The first P. A. M. C. shall be held in the city of Washington, D. C., Sept. 5, 6, 7, 8, A. D., 1893.

The words "Medical Profession" in the "General Regulation" are intended to include the dental profession on the same ground that they are entitled to membership in the American Medical Association, viz.: That one holding the title of D.D.S., without the M.D., is eligible to membership, consequently it is expected that the section of Oral and Dental Surgery will form one of the most interesting sections of the Congress.

